# Retroviruses and retroelements in diseases and in gene therapy: 15 years later

**DOI:** 10.1186/1750-9378-6-14

**Published:** 2011-09-24

**Authors:** Jan Svoboda

**Affiliations:** 1 Institute of Molecular Genetics AS CR, Vídenská 1083, 142 20 Prague 4, Czech Republic

## Abstract

The past 15 years opened new avenues for retrovirus and retroelement research. Not surprisingly, they stemmed from essential knowledge collected in the past, which remains the ground of the present and therefore should be remembered. However, a short supplement of new break-through discoveries and ideas should be recollected. Using selected examples of recent works, I tried to extend and supplement my original article published in Folia Biologica (1996).

## Retrovirus integration

**Figure 1:**
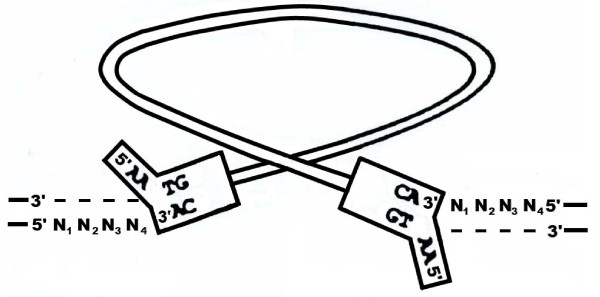
**Scheme of retrovirus integration (modified from Goff 1992)**[30]. Note that linear viral DNA integrates in antiparallel host cell DNA strands. Ends of viral DNA comprising critical part of long terminal repeats are boxed. N denotes nucleotide- complementary nucleotide. A full description of integration steps is given in the text.

Having an insight into the molecular events governing the central process of retrovirus replication cycle represented by reverse transcription, the question of site specificity of retrovirus DNA integration became the focus of interest. In order to introduce the process of retrovirus integration the main steps involved in its realization are briefly summarized (see Figure 1) Viral DNA synthesized by reverse transcription is recessed at both ends constituted of long terminal repeats (LTR). Such cleavage leads to the formation of CA dinucleotide overhangs. The next step is accomplished by staggered nick on host DNA and joining 3' recessed ends of viral DNA with 5' end nucleotides of host cell DNA. The non-complementary dinucleotides AA from the 5' ends of viral DNA are then removed and the gaps between protruding 5' ends of cleaved host cell DNA are filled by DNA repair leading to formation of several nucleotide repeats flanking the integrated provirus [1].

At present, new knowledge about the interaction of viral integrase with viral DNA has been obtained using protein crystallography (for references see Li et al., 2011)[2]. Integrase acts as a tetramer and engages both viral DNA ends. It triggers formation of recessed end joining of 3' end of viral with 5' end of cell DNA. These activities depend on a series of cellular factors; one of them, the lens epithelium-derived growth factor (LEDGF), targets HIV DNA, preferentially at active transcription units. LEGDF acts as an adaptor equipped with integrase and chromatin-binding domains. The latter can be substituted with sequences binding other proteins which lead to preferential provirus targeting to selected genome regions [3]. Further knowledge of processes deciding about provirus positioning in the cell genome will undoubtedly contribute to designing of safe retroviral vectors.

There are clear differences in the affinity of retroviruses to certain cell genomic regions. ALU/ASV tend to integrate in GC-rich, preferentially house-keeping genes [4,5]. A similar situation is encountered in HIV infection [6]. However, mouse leukemia virus (MLV) integrates close to gene promoters, which facilitates cell gene activation [7]. If a protooncogene is activated in such a way, it can trigger tumor formation, as was documented in humans infected with MLV-based retroviral vector resulting in LMO oncogene activation and lymphoma formation [8].

## Update of retroviral vector construction

Understanding the conditions under which retrovirus integration can be directed in defined cell genome regions has a principal significance for construction of safe and long-term expressed vectors. This goal seems to be close, but still requires careful testing.

Detailed study of representative retroviruses indicates that two of them represent the best candidates for vector construction. The first are lentiviruses, among which belongs HIV and similar complex viruses isolated from different mammalian species. These viruses are pathogenic but not oncogenic. They are equipped with a series of accessory genes that also enable their integration in non-dividing cells. Not surprisingly, lentiviruses stripped of their pathogenic sequences were successfully employed for construction of vectors. It was reported that they also acted as an efficient tool for gene therapy of adrenoleukodystrophy (ALD) [9]. ADL is caused by mutation of a peroxisomal transporter gene and its deficiency leads to demyelination and consequently to the nervous system dysfunction. The undamaged gene was transduced by a lentiviral vector to autologous hematopoietic stem cells, which were then implanted to ADL patients. Encouraging results were obtained because about 10% patients' granulocytes expressed the transmitted gene for more than two years and the neurological function became stabilized. Further good news is provided by the clonality of the transduced cells, suggesting random vector integration without striking overgrowth of clonal populations, which might signal a carcinogenic change.

The second candidates for safe vectors are provided by ALV/ASV avian retroviruses. These retroviruses do not replicate in mammalian cells, and therefore it is not required to strip them of replicative genes in order to prevent their multiplication. Furthermore, ALV sequences are not homologous to human endogenous retroviral genomes and therefore there is almost no chance of their mutual recombination. However, ALV integrated in mammalian cells are usually underexpressed and prone to silencing by methylation. Nevertheless, retroviral vectors can be protected against silencing by insertion of CpG islands close to their promoter [10]. Such and additional improvements should increase their chance to become safe candidates for vector construction.

## Retrotransposable elements

Retrotransposable elements, which employ reverse transcription for their genesis and even for their spread through the genome, became an important issue of genomics, evolutionary genetics, oncology, and other fields of general interest.

We now know that more than 45% of our genome arose by reverse transcription, which includes 8% of proviral sequences and 18% of mobile LINE elements that are involved in spreading the gene sequences and responsible for pseudogene formation. In contrast to retroviruses LINE elements use as primers for reverse transcription stretches of oligo-dT. Such dT rich regions can aneale with with polyA tails of cell RNAs. Therefore in some cases a mRNA substitutes LINE RNA template becomes reverse transcribed to DNA constituting intronless pseudogenes In spite of the fact that LINE are mostly defective, about hundred LINE copies remain active and retrotranspose to our genome.

**Figure 2:**
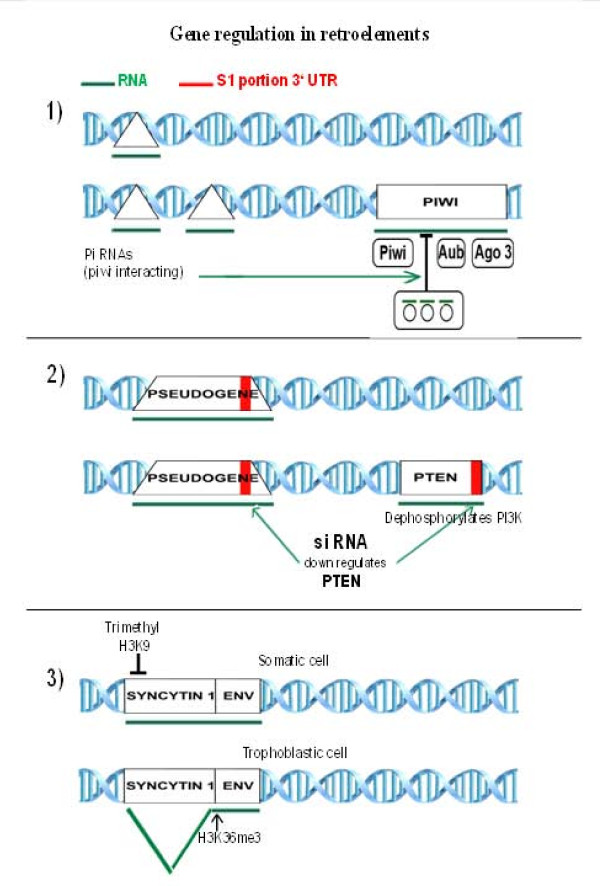
**Examples of retroelement regulation and its ability to regulate other genes.** Protein products are encircled. Syncytin 1 gene corresponding to endogenous retroviral structure is presented in two parts. The left covers the retroviral sequence and the right part depicts retroviral envelope gene (env). For explanation see text.

The field of retrotransposable elements had been reviewed several times in the past, but sophisticated cell control over these elements was recently summarized by Goodier and Kazazian, 2008[11]; Blumenstiel, 2011[12]. In order to illustrate some of present-day topics that are being analyzed in depth, the following three examples will be shortly discussed (Figure 2).

The first example is represented by PIWI elements, which spread in Drosophila and rapidly expanded in mammalian evolution [13]. PIWI spreading is counteracted by piRNAs, whose interaction with PIWI proteins leads to inhibition of PIWI transcription. This process is even more complicated due to different effects of sense or antisense piRNA and it can lead to amplification of signals inhibiting PIWI transcription[14,15] (see Figure 2). Drosophila piRNA clusters were identified as ancient fragmented transposon copies, however being significantly diverged from active retrotransposon sequences (reviewed in Malone and Hannon, 2009)[16].

The second case (Figure 2) documents how pseudogenes can regulate very important tumor suppressor gene PTEN, which inhibits the oncogene PI3K by dephosphorylation. PTEN is under the control of small interfering RNA (siRNA), which down-regulates its activity. However, such siRNA also binds to the S1 region of 3' UTR retained in PTEN pseudogenes. In such a way pseudogenes saturate siRNA and thus potentiate the activity of the PTEN tumor suppressor gene [17].

The third example (Figure 2) relates to human endogenous retrovirus belonging to the HERV-W family. In humans there are two members of this family causing cell fusion resulting in formation of syncytiotrophoblast. They are called syncytin 1 and 2, both producing retroviral glycoprotein responsible for cell fusion. Syncytin 1(belonging to HERV-W group) is down-regulated in somatic cells by the lack of splicing resulting in the absence of viral envelope mRNA and trimethylation of histone 3K9. On the contrary, in trophoblastic cells the syncytin message is efficiently spliced and methylation is also changed in favor of the methylation pattern potentiating splicing (H3K36) [18]. This example illustrates how proviruses can be employed to perform important functions in the organism and how they become subdued to cell regulation and utilized for new cell functions.

The already mentioned three examples of retroelement involvement in cell functions do not cover the full extent of their activities. There is growing evidence that retroelements take part in the control of gene expression. Evidence has been presented showing significantly increased retroelement representation in the promoter region of genes, especially those involved in development, cell differentiation and transcription regulation, and also in additional structures delineating functional genes such as locus control regions (LCR) or insulators acting as a divide of individual enhancer. The sphere of influences was reviewed by King Jordan et al., 2003[19]; Lowe et al., 2007[20]. Thus, to understand gene regulation, we must take into account also retroelements.

Of special interest is the evolution of retroelements and their role in the constitution of new important cell functions. The evolutionary role of retroelements is strongly supported by several observations documenting that some of them have been conserved during evolution of tetrapods and therefore they should have served functions not defined so far [21]. Needless to say many other retroelements were probably modified to such an extent that at present they escape identification. However, there are available some instances documenting clearly that retroelements fulfill a key function. One of them pertains to telomerase function, which serves for extension of telomeric nucleotides, thus sealing gaps arising at 5' ends of the replicating DNA strand. In lower multicellular organisms such as Rotifera or some plants, telomere is extended by its annealing with retroposon Penelope equipped with telomere hexanucleotide repeats, which reconstitute telomere during reverse transcription [22].

Good statistical evidence is available demonstrating that the human genome contains more Alus than the chimp genome. More sensitive techniques provide evidence for more extensive representation of retroelements, namely Alu and LINE, estimated as 10^6 ^insertions in individual human genomes worldwide [23,24]. Of special interest are the findings of retroelement involvement in neural tissue differentiation. In relation to this topic I would like to comment on the discovery of a highly conserved SINE element (LF-SINE) detected already in Latimeria (from Silurian period 410 million years ago), which remained at the same position during later evolution [21]. As proven experimentally, this element acts as an enhancer of neurodevelopmental gene (ISL1), which is required for motor neuron differentiation. Studies of microcephalin gene involved in the establishment of human brain revealed that it became a target of thousands of retrotransposable element insertions and that these elements constitute 57% of gene length [25,26]. Recent observations support the possibility that at least L1 retrotransposition in rat and human neural tissues might play a role in producing functional imprints on individual neural cells [27].

Looking at retroelements from a more general point of view, we should consider their ability to spread not only vertically, but also horizontally. This was encountered at first in LTR containing retroelement copies that were shown to be transmitted among different species of Drosophila. Similarly, LINE elements might have been transferred between snakes and mammals [28] Such horizontal transfers cannot be regarded only as odd situations, because any distant species transfer provides retroelement relief from original cell control and enables new possibilities of spreading [29].

In summary, retroelements should be taken seriously not only as principal factors reshaping any genome, but also as elements that due to their mobility can cause damage contributing to the occurrence of important diseases.

## Competing interests

The author declares that he has no competing interests.
